# Totally Endoscopic Internal Thoracic Artery Harvesting with Efficient Setup Modifications for Minimally Invasive Direct Coronary Artery Bypass Grafting

**DOI:** 10.5761/atcs.nm.25-00007

**Published:** 2025-03-07

**Authors:** Yoshihiro Goto, Yui Ogihara, Sho Takagi, Junji Yanagisawa, Yasuhide Okawa

**Affiliations:** Department of Cardiovascular Surgery, Toyohashi Heart Center, Toyohashi, Aichi, Japan

**Keywords:** MIDCAB, CABG, thoracoscopy, internal thoracic artery, minimally invasive cardiothoracic surgery

## Abstract

Minimally invasive direct coronary artery bypass grafting for the left anterior descending artery is a well-established technique; however, harvesting the internal thoracic artery is challenging, particularly with endoscopic approaches. In this study, 12 patients underwent internal thoracic artery harvesting using a three-dimensional endoscope with a three-port system (one incision plus two ports). Working space was established by elevating the chest wall upward using hooks anchored at the main incision site. To enhance operability, the positions of the camera and instruments were strategically adjusted within the existing ports, obviating the need for additional access points. All patients achieved graft patency. No complications, such as internal thoracic artery injury, were observed, and no patient required conversion into median sternotomy. This approach minimizes invasiveness while maintaining effectiveness, allowing for adequate dissection of the internal thoracic artery without necessitating expansion of the existing surgical setup.

## Introduction

Although favorable outcomes of coronary artery bypass grafting via median sternotomy have been reported even in older patients, careful consideration is required for cases with high preoperative risk.^1)^

The potential for reducing patient burden makes minimally invasive direct coronary artery bypass grafting (MIDCAB), which targets the left anterior descending artery (LAD), an established therapeutic option.^2)^ However, harvesting the internal thoracic artery (ITA) endoscopically is more challenging than harvesting it via a median sternotomy. For MIDCAB, ITA harvesting can be performed using direct visualization or via total endoscopic methods, including robot-assisted techniques. Thoracoscopic surgery without robotic assistance is less expensive but has lower operability and requires a high level of proficiency. In addition, the morphologies of the chest and heart can complicate ITA harvesting. The optimal setup positions vary across institutions, and no standardized method has been established.^2,3)^ Here, we report a novel technique that improves operability and allows safe and extensive ITA harvesting under total endoscopy while maintaining minimal invasiveness by optimizing the use of the camera and instruments within existing ports.

## Case Report

**Fig. 1:**
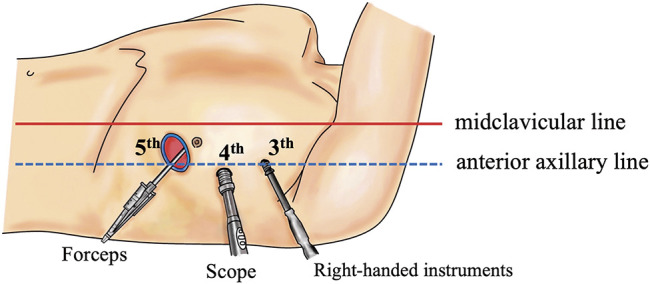
Surgical setting. Schema of instrument distribution to the three ports.

**Fig. 2:**
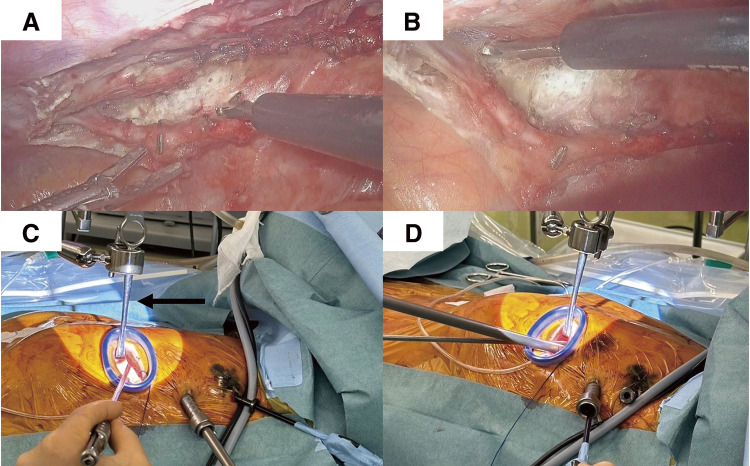
(**A**) Endoscopic view of the distal ITA site from the standard position. (**B**) Endoscopic view of the distal ITA site from the modified position. (**C**) Photograph of the standard surgical setup. (**D**) Photograph of the modified surgical setup. Arrow: traction is applied at the primary incision using hooks to increase the working space. ITA: internal thoracic artery

**Fig. 3:**
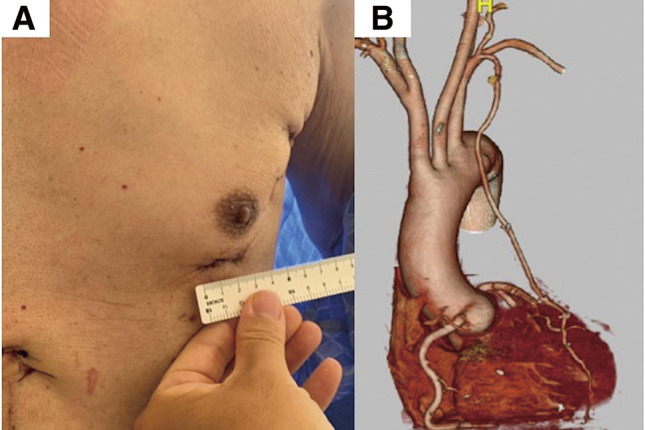
(**A**) Postoperative wound. (**B**) Postoperative contrast-enhanced CT confirms graft patency. CT: computed tomography

Preoperative contrast-enhanced computed tomography (CT) was performed on 12 consecutive patients to assess the LAD anastomosis sites and to confirm the ITA course. All surgical procedures were performed under single-lung ventilation, with patients positioned in a 30° right-side rotation and the left upper limb elevated. A 3–5 cm skin incision was made laterally from the midline of the clavicle at the fifth intercostal space, and a 10-mm camera port and a 5-mm port were created at the anterior axillary line in the fourth and third intercostal spaces, respectively (**Fig. 1**). The ITA was harvested using a three-dimensional (3D) endoscope (Karl Storz, Tuttlingen, Germany) and a skeletonized technique using a harmonic scalpel. The 3D camera, dissecting instrument, and forceps were inserted through the 10-mm port, 5-mm port, and main incision, respectively. A long-shaft harmonic device was used through the 5-mm port. In cases where distal ITA harvesting was challenging because of the chest morphology, the setup was modified. A hook was used at the main incision to lift the chest wall upward and secure the working space. Instruments for the left hand and the camera were inserted through the main incision, and the dissecting tool was moved to the 10-mm port previously used for the camera (**Fig. 2**). The 3D endoscope used in this study featured a 180° image rotation capability, allowing adjustments to mitigate instrument interference during surgical setup changes. In addition, we used forceps with a large tip bending angle to prevent interference between the forceps shaft and the camera. These adjustments further improved operability and minimized procedural complexity. The ITA was harvested from the first rib branch proximally to the branching point distally. Heparin (150 units/kg) was administered, and the pericardium was incised to identify the LAD. The Octopus Nuvo stabilizer (Medtronic Inc., Minneapolis, MN, USA) was inserted via the camera port or near the subxiphoid region to stabilize the heart. Side-to-side anastomosis was performed through the main incision using 8-0 polypropylene sutures. In one patient, the target site was higher, requiring an additional approach through the same skin incision at the fourth intercostal space. Protamine was administered after anastomosis, hemostasis was confirmed endoscopically, and the procedure was completed (**Video**). No patient experienced ITA injury or required conversion to a median sternotomy. The mean procedure time was 177 ± 33 min, with an ITA harvesting time of 63 ± 12 min. Postoperative pain was managed with oral acetaminophen, administered to eight patients, with a total dose of 2200 mg (range: 1000–3000 mg). Graft patency was confirmed in all patients using contrast-enhanced CT (**Fig. 3**). No re-exploration was required, and all patients were discharged home after a median of 5 days (range, 3–10 days) postoperatively.

## Discussion

MIDCAB has been a well-established treatment for LAD lesions since its introduction in 1967. The left ITA bypass is the preferred choice due to its excellent outcomes.^4)^ A key aspect of MIDCAB is ITA harvesting, which can be performed using direct visual or endoscopic approaches. The direct visual approach, while commonly involving access through the fourth intercostal space, often requires a larger incision owing to limited visibility, complicating adequate ITA harvesting.^2,3)^ In contrast, endoscopic techniques offer advantages such as reduced postoperative pain and better cosmetic outcomes but demand specialized surgical expertise.^3)^ Although robotic assistance significantly enhances surgical precision, its high cost restricts widespread accessibility. Various techniques have been described for non-robotic endoscopic procedures; however, the optimal configuration for camera and port placement varies among institutions and is debated.^2,3)^ Additionally, anatomical challenges such as cardiac enlargement or chest-wall deformities further complicate effective ITA harvesting. Although CO_2_ insufflation improves visualization, caution is required due to potential hemodynamic changes associated with open pneumothorax. Additional ports are sometimes necessary for setup adjustments.^5)^ Issues like insufficient graft length or arterial damage, although rare, may necessitate conversion to a median sternotomy. Side-to-side anastomosis demonstrated good hemodynamics and was feasible with adequate ITA length.^6)^ To enhance surgical maneuverability while avoiding the introduction of additional ports, a hook was strategically placed over the primary wound to elevate the chest wall, thereby improving visual access and operative precision. A technique involving an additional incision in the subxiphoid region and chest wall elevation has been reported.^7)^ However, we applied controlled traction directly through the primary incision without expanding the intercostal space, thereby minimizing the risk of bleeding and reducing postoperative pain. Camera positioning was also refined to improve safety and operability, drawing on methods employed in extensive endoscopic surgeries, such as those for esophageal cancer.^8)^ Increasing the number of ports in endoscopic surgery may improve maneuverability; however, it intensifies pain and increases the length of hospital stay.^9)^ Our approach uses a streamlined three-port system, comprising one small incision and two additional ports, enabling efficient ITA harvesting without the need for supplemental access points. Shifting the entire surgical setup caudally by one rib ensured consistent frontal visualization of the distal ITA and its branches, eliminating blind spots and enhancing operative precision. To ensure effective adjustment, positioning the camera port in a manner that allows for optimal manipulation of instruments with the right hand is critical. In thoracoscopic surgery, the camera should be positioned between the right- and left-hand ports to ensure optimal visualization and instrument coordination.^10)^ Our method adheres to this principle, even after modification of the surgical setup.

## Conclusions

We reported a technique that supports extensive, cost-effective ITA harvesting while preserving the cosmetic and pain-management benefits. In situations where operability and visualization are limited, procedural safety and efficiency can be further optimized by modifying instrument placement and manipulation techniques or by using chest-wall traction to expand the surgical field. We believe this method offers significant advantages in endoscopic ITA harvesting and contributes to enhanced surgical outcomes in MIDCAB.

## Declarations

### Ethics approval and consent to participate

Toyohashi Heart Center Ethics Committee (Approval ID: THC240311).

### Consent for publication

Not applicable.

### Funding

Not applicable.

### Disclosure statement

The author declares there is no conflict of interest related to this work.

### Data availability

Not applicable.

### Author contributions

YG: responsible for study conception, surgical procedures, and drafting the manuscript.

YO: contributed to data collection and analysis.

ST: contributed to data collection and analysis.

JY: contributed to data collection and analysis.

YO: supervised the project and critically reviewed the manuscript.

All authors have read and approved the final manuscript.

## Supplemental Information

Video Intraoperative video demonstrating totally endoscopic internal thoracic artery harvesting with optimized setup adjustments for minimally invasive direct coronary artery bypass grafting.
